# Nomogram Combining Radiomics With the American College of Radiology Thyroid Imaging Reporting and Data System Can Improve Predictive Performance for Malignant Thyroid Nodules

**DOI:** 10.3389/fonc.2021.737847

**Published:** 2021-10-13

**Authors:** Xingzhi Huang, Zhenghua Wu, Aiyun Zhou, Xiang Min, Qi Qi, Cheng Zhang, Songli Chen, Pan Xu

**Affiliations:** ^1^ Department of Ultrasonography, The First Affiliated Hospital of Nanchang University, Nanchang, China; ^2^ Department of Head and Neck Otolaryngology, The First Affiliated Hospital of Nanchang University, Nanchang, China

**Keywords:** thyroid nodule, radiomics, nomogram, ultrasound, prediction

## Abstract

**Purpose:**

To develop and validate a nomogram combining radiomics of B-mode ultrasound (BMUS) images and the American College of Radiology (ACR) Thyroid Imaging Reporting and Data System (TI-RADS) for predicting malignant thyroid nodules and improving the performance of the guideline.

**Method:**

A total of 451 thyroid nodules referred for surgery and proven pathologically at an academic referral center from January 2019 to September 2020 were retrospectively collected and randomly assigned to training and validation cohorts (7:3 ratio). A nomogram was developed through combining the BMUS radiomics score (Rad-Score) with ACR TI-RADS score (ACR-Score) in the training cohort; the performance of the nomogram was assessed with respect to discrimination, calibration, and clinical application in the validation and entire cohorts.

**Results:**

The ACR-Rad nomogram showed good calibration and yielded an AUC of 0.877 (95% CI 0.836–0.919) in the training cohort and 0.864 (95% CI 0.799–0.931) in the validation cohort, which were significantly better than the ACR-Score model (*p* < 0.001 and 0.031, respectively). The significantly improved AUC, net reclassification index (NRI), and integrated discriminatory improvement (IDI) of the nomogram were found for both senior and junior radiologists (all *p* < 0.001). Decision curve analysis indicated that the nomogram was clinically useful. When cutoff values for 50% predicted malignancy risk (ACR-Rad_50%) were applied, the nomogram showed increased specificity, accuracy and positive predictive value (PPV), and decreased unnecessary fine-needle aspiration (FNA) rates in comparison to ACR TI-RADS.

**Conclusion:**

The ACR-Rad nomogram has favorable value in predicting malignant thyroid nodules and improving performance of the ACR TI-RADS for senior and junior radiologists.

## Introduction

With the increasing number of imaging-detected thyroid nodules, overdiagnosis and overtreatment are major clinical challenges in the management of these nodules; therefore, an accurate and practical risk stratification tool is necessary (1). Because B-mode ultrasound (BMUS) is the most accurate imaging modality to assess thyroid nodules, there are a number of risk classification systems based on BMUS images formulated by authoritative associations (2–5). Previous studies have compared different guidelines to find a management guideline that is most beneficial to patients and demonstrated that the 2017 American College of Radiology (ACR) Thyroid Imaging Reporting and Data System (TIRADS) showed accurate diagnostic performance and meaningful reduction in the number of thyroid nodules recommended for biopsy (6–8). However, the relatively low specificity, positive predictive value (PPV), and interobserver variability of the ACR guidelines are impediments to achieving the desired clinical results (7, 9).

Radiomics has the ability to high-throughput mine quantitative image features and discover information reflecting the underlying pathophysiology that cannot be assessed by visual interpretation (10, 11). In recent years, radiomics has been applied to the thyroid, showing that it helps predict malignancy in thyroid nodules and preoperative cervical lymph node staging in papillary thyroid carcinoma (12–14). However, radiomics features are usually analyzed from a single-section image of the target nodule; therefore, radiomics alone might lose some important BMUS information, which makes it impossible to significantly improve the performance of risk stratification systems for all radiologists with different proficiency levels (15).

A nomogram is a graphical tool for a concise and intuitive display of the predicted value of individual outcome events based on multivariate regression analysis. We supposed that a nomogram could adequately combine the visual interpretation and radiomics of BMUS images to achieve better predictive performance. To the best of our knowledge, no published study has investigated the predictive performance of a nomogram combining radiomics with ACR TI-RADS scores for predicting malignant thyroid nodules.

Therefore, the purpose of our study was to develop and validate a nomogram that combines the radiomics score (Rad-Score) and ACR-TIRADS score (ACR-Score) for predicting malignant thyroid nodules and improving performance of the ACR TI-RADS.

## Materials and Methods

Ethical approval and informed consent were waived because the retrospective study with de-identified data was used, and no protected health information was needed. The study was conducted following guidelines by the Declaration of Helsinki.

### Patients

Between January 2019 and September 2020, patients with thyroid nodules (≥10 mm in maximum diameter) in the Head and Neck Otolaryngology Department of our institution were consecutively included. The nodules were enrolled using the following inclusion and exclusion criteria.

The inclusion criteria were as follows: 1) the target nodule had undergone surgical resection; 2) postoperative pathological results were obtained; and 3) BMUS was performed within 2 weeks before the resection. Exclusion criteria are as follows: 1) the pathological result of the nodule was ambiguous, 2) interventional procedures such as fine-needle aspiration (FNA) and radiofrequency ablation were performed before BMUS, and 3) the BMUS image of the target nodule was unclear.

A total of 451 patients (median age, 45 years, range, 20 to 81 years; 93 men and 358 women) were enrolled. If there were multiple nodules in one patient, the nodule with the largest diameter was selected as the target nodule. All nodules were randomly split into a training cohort (n = 315, median age, 45 years, range 20 to 81 years; 68 men and 247 women) and a validation cohort (n = 136, median age, 43 years, range 21 to 70 years; 25 men and 111 women) in a 7:3 ratio.

### Clinical and BMUS Information

Clinicopathological data, including age, sex, and nodule pathology, were obtained from medical records. BMUS images were acquired with a Philips iU Elite and Philips EPIQ7 (ultrasound system, Philips Medical System, Bothell, WA, USA) using a 5–12-MHz linear transducer by two radiologists (PX and ZW) with more than 8 years of experience. Images of each target nodule were obtained in transverse and longitudinal planes, and video clips were obtained in at least one plane.

### Analysis of the ACR TI-RADS

Two radiologists (AZ and XH, with more than 10 years and 3 years of experience, respectively) who were unaware of the pathological results reviewed the BMUS images of all nodules. The five feature categories in the ACR TI-RADS lexicon (composition, echogenicity, shape, margin, and echogenic foci) were evaluated, and the ACR-Score of each nodule was calculated (referred to as ACR-Score 1 for AZ, ACR-Score 2 for XH) (16). The Supplement presents the detailed process of calculating the ACR-Score (**Supplementary Tables E1**, **2**). The diameter and location (subcapsular or intrathyroidal) were negotiated to a consensus by the two radiologists.

### Analysis of the Radiomics Features

**Figure 1:**
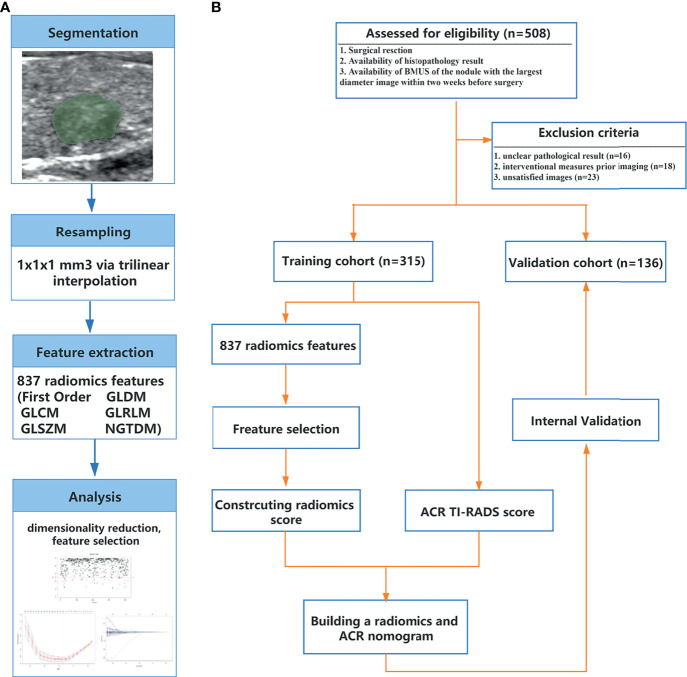
B-model ultrasound radiomics workflow **(A)** and study flowchart **(B)**. ACR, American College of Radiology; BMUS, B-model ultrasound; GLCM, gray-level co-occurrence matrix; GLDM, gray-level dependence matrix; GLRLM, gray-level run length matrix; GLSZM, gray-level size zone matrix; NGTDM, neighboring gray tone difference matrix; TI-RADS, thyroid imaging reporting and data system.

The region of interest (ROI) was delineated manually on the BMUS DICOM image of the target nodule with the largest diameter in sagittal view using open-source software (3D Slicer, version 4.10.2; https://www.slicer.org) (**Supplementary Figure E1**) (17, 18). The reproducibility of the intra- and interobserver agreement for the radiomics features was measured using the first 130 nodules that a radiologist (XH) redelineated twice within 2 weeks. The intraclass correlation coefficient (ICC) was used to evaluate the intra- and interobserver agreement. ICC > 0.75 represented satisfactory agreement. XH delineated the remaining nodules if strong agreement (ICC > 0.90) was achieved. To ensure repeatability of the results, resampling and z-score normalization were performed as preprocessing steps (**Supplementary E1**). Open-source software (Pyradiomics; http://pyradiomics.readthedocs.io/en/latest/index.html) (19) was used to extract a total of 837 texture, intensity, and wavelet features (**Supplementary Table E3**). Then, dimensionality reduction and radiomics feature selection were performed successively by ICC, univariate, least absolute shrinkage and selection operator (LASSO) and linear dependence analyses (20, 21). The methodology used to extract the radiomics features is further described in **Supplementary E2** and **Figure 1A**. The radiomics score (Rad-Score) was generated using a linear combination of the selected features.

### Statistical Analysis

Statistical analysis was conducted with IBM SPSS 22.0 software (IBM, New York, USA) and R software (Version 4.0.1, https://www.r-project.org/). The packages of R4.0.1 used are provided in **Supplementary Table E4**. The Shapiro–Wilk test was used to evaluate the normality of the distribution. Continuous data conforming to a normal distribution are expressed as the mean ± standard deviation (SD) and were compared using Student’s t-test; nonconforming data are expressed as the median [interquartile range (IQR)] and were compared using the Mann–Whitney U test. Categorical data are expressed as numbers (%) and were compared using a chi-square test or Fisher’s exact test, as appropriate. A *p* < 0.05 represented a statistically significant difference.

#### Development of the ACR-Rad Nomogram

The ACR-Rad nomogram was developed based on the Rad-Score and the average of ACR-Score 1 and ACR-Score 2. For comparison, the ACR-Score model was built through a univariate logistic equation.

#### Performance of the ACR-Rad Nomogram

Calibration was evaluated using the Akaike information criterion (AIC), the Bayesian information criterion (BIC), calibration curve, and Hosmer–Lemeshow test (22). Discrimination performance was evaluated using the area under the receiver operator characteristic (ROC) curve (AUC). The Delong test was used to compare AUCs and the LR test to compare the effect across the nested logistic regression models.

#### Clinical Utility of the ACR-Rad Nomogram

The interobserver agreement for ACR-Score, Rad-Score, and the predicted malignancy risk by the ACR-Rad nomogram was evaluated. The improvement in the predictive accuracy of the nomogram by radiologists with different levels of experience was evaluated by the AUC, index integrated discrimination improvement (IDI), and net reclassification improvement (NRI). Decision curve analysis (DCA) was conducted to determine the clinical usefulness of the nomogram by quantifying the net benefits at different threshold probabilities in the entire cohort.

For clinical management, we compared the performance for biopsy recommended of the cutoff value which was determined in the training cohort with the maximum Youden index (referred to as ACR-Rad_max), and different cutoff values which were determined in the training cohort for prespecified predicted risks of malignancy (20%/30%/40%/50%) (referred to as ACR-Rad_20%/30%/40%/50%, respectively) with ACR TI-RADS in the entire cohort.

## Results

### Patient Characteristics

The study flowchart is shown in **Figure 1B**. Clinical and pathological characteristics in the training and validation cohorts are summarized in **Tables 1**, **2**. There was no significant difference between the training and validation cohorts for clinicopathological and BMUS characteristics (all *p* > 0.05). The proportions of malignant nodules in the two groups were 62.9% (198/315) and 70.6% (96/136) (*p* = 0.114). Malignant nodules had significantly lower age, diameter, and nodular goiter and significantly higher ACR-Score 1 and ACR-Score 2 than benign nodules in the training and validation cohorts (all *p* < 0.05).

**Table 1:** Clinicopathological and ultrasonic characteristics of patients in the training and validation cohorts.

	Entire population (n = 451)	Training cohort (n = 315)	Validation cohort (n = 136)
Sex			
Male	93 (20.6)	68 (21.6)	25 (18.4)
Female	358 (79.4)	247 (78.4)	111 (81.6)
Age, years	45 (34-53)	45 (32-53)	45 (37-53)
Diameter, mm	15.0 (13.0-19.5)	15.0 (13.0-19.5)	15.9 (13.0-19.5)
Nodule pathology			
Benign	157 (34.8)	117 (37.1)	40 (29.4)
Malignant	294 (65.2)	198 (62.9)	96 (70.6)
Tumor location			
Subcapsular thyroid	319 (70.7)	221 (70.2)	98 (72.1)
Intra-thyroidal	132 (29.3)	94 (29.8)	38 (27.9)
Hashimoto thyroiditis			
Positive	162 (35.9)	113 (35.9)	49 (36.0)
Negative	289 (64.1)	202 (64.1)	87 (64.0)
Nodular goiter			
Positive	154 (34.1)	112 (35.6)	42 (30.9)
Negative	297 (65.9)	203 (64.4)	94 (69.1)
ACR-Score 1*^b, c^*	7 (5-9)	8 (5-9)	7 (5-9)
Composition			
Cystic or spongiform	21 (4.7)	14 (4.4)	7 (5.1)
Cystic and solid	54 (12.0)	41 (13.0)	13 (9.6)
Solid	376 (83.4)	260 (82.5)	116 (85.3)
Echogenicity			
Anechoic	21 (4.7)	14 (4.4)	7 (5.1)
Hyper- or isoechoic	82 (18.2)	65 (20.6)	18 (13.2)
Hypoechoic	277 (61.4)	189 (60.0)	87 (64.0)
Very hypoechoic	71 (15.7)	47 (14.9)	24 (17.6)
Shape			
Taller-than-wide	132 (29.3)	92 (29.2)	40 (29.4)
Not taller-than-wide	319 (70.7)	223 (70.8)	96 (70.6)
Margin			
Smooth or ill defined	163 (36.1)	118 (37.5)	45 (33.1)
Irregular or lobulated	281 (62.3)	193 (61.3)	88 (64.7)
Extrathyroidal extension	7 (1.6)	4 (1.3)	3 (2.2)
Echogenic foci*^a^*			
No echogenic foci or large comet tail	233 (51.7)	162 (51.6)	71 (51.4)
Macrocalcifications	46 (10.2)	29 (9.2)	17 (12.3)
Peripheral	8 (1.8)	5 (1.6)	3 (2.2)
Punctate	165 (36.6)	118 (37.6)	47 (34.1)
ACR TI-RADS risk level			
TR1	21 (4.7)	14 (4.4)	7 (5.1)
TR2	19 (4.2)	15 (4.8)	5 (3.7)
TR3	31 (6.9)	26 (8.3)	5 (3.7)
TR4	103 (22.8)	69 (21.9)	34 (25.0)
TR5	277 (61.4)	191 (60.6)	85 (62.5)
ACR-Score 2*^c^*	8 (6-9)	8 (6-9)	8 (6-9)
Rad-Score	0.910 (-0.100-1.550)	0.932 (-0.038-1.694)	0.899 (-0.114-1.473)

Qualitative data were expressed as mean ± standard deviation or number and percentages (%); quantitative data were expressed as median (25%–75% quantiles).

ACR, American College of Radiology; TI-RADS, Thyroid Imaging Reporting and Data System.

^a^Nodules could have more than one type of echogenic foci.

^b^B-model ultrasound findings based on the senior interpretation.

^c^ACR-Score 1 was referred for the senior radiologist, ACR-Score 2 for the junior radiologist.

**Table 2:** Clinicopathological and ultrasonic characteristics for thyroid nodules in the training and validation cohorts by pathology.

Characteristic	Training cohort			Validation cohort		
	Benign (n = 117)	Malignant (n = 198)	*p* value	Benign (n = 40)	Malignant (n = 96)	*p* value
Sex			0.942			0.511
Male	25 (21.4)	43 (21.7)		6 (24.0)	19 (30.6)	
Female	92 (78.6)	155 (78.3)		34 (76.0)	77 (69.4)	
Age, years	47 (38-55)	42.5 (30-50)	0.001	51.5 (43.5-60)	42.5 (33.5-49)	<0.001
Diameter, mm	17.0 (14.0-32.0)	15.0 (12.0-17.0)	<0.001	19.5 (14.0-34.0)	15.0 (13.0-17.0)	<0.001
Tumor location			0.816			0.236
Subcapsular thyroid	83 (70.9)	138 (69.7)		26 (65.0)	72 (75.0)	
Intra-thyroidal	34 (29.1)	60 (30.3)		14 (35.0)	24 (25.0)	
Hashimoto thyroiditis			0.470			0.344
Positive	39 (33.3)	74 (37.4)		12 (30.0)	37 (38.5)	
Negative	78 (66.7)	124 (62.6)		28 (70.0)	59 (61.5)	
Nodular goiter			0.003			0.021
Positive	54 (46.2)	58 (29.3)		18 (45.0)	24 (25.0)	
Negative	63 (53.8)	140 (70.7)		22 (55.0)	72 (75.0)	
ACR-Score 1*^b, c^*	4 (3-7)	9 (7-9)	<0.001	5 (2-6)	8 (6-10)	<0.001
Composition			<0.001			<0.001
Cystic or spongiform	14 (12.0)	0 (0)		7 (17.5)	0 (0)	
Cystic and solid	32 (27.4)	9 (4.5)		10 (25.0)	3 (3.1)	
Solid	71 (60.7)	189 (95.5)		23 (57.5)	93 (96.9)	
Echogenicity			<0.001			<0.001
Anechoic	14 (12.0)	0 (0)		7 (17.5)	0 (0)	
Hyper- or Isoechoic	51 (43.6)	14 (7.1)		14 (35.0)	4 (4.2)	
Hypoechoic	35 (29.9)	154 (77.8)		17 (42.5)	70 (72.9)	
Very hypoechoic	17 (14.5)	30 (15.2)		2 (5.0)	22 (22.9)	
Shape			<0.001			0.017
Taller-than-wide	18 (15.4)	74 (37.4)		6 (15.0)	34 (35.4)	
Not taller-than-wide	99 (84.6)	124 (62.6)		34 (85.0)	62 (64.6)	
Margin			<0.001			<0.001
Smooth or ill defined	87 (74.4)	42 (20.1)		29 (72.5)	16 (16.7)	
Irregular or lobulated	29 (24.8)	164 (78.5)		11 (27.5)	77 (80.2)	
Extrathyroidal extension	1 (0.9)	3 (1.4)		0 (0)	3 (3.1)	
Echogenic foci*^a^*			<0.001			0.028
No echogenic foci or Large comet tail	75 (64.1)	87 (43.5)		25 (59.5)	46 (47.4)	
Macrocalcifications	18 (15.4)	11 (5.5)		8 (19.0)	9 (9.3)	
Peripheral	2 (1.7)	3 (1.5)		2 (4.8)	2 (2.1)	
Punctate	22 (18.8)	99 (49.5)		7 (16.7)	40 (41.2)	
ACR TI-RADS risk level			<0.001			<0.001
TR1	14 (12.0)	0 (0)		7 (17.5)	0 (0)	
TR2	14 (12.0)	1 (0.5)		4 (10.0)	1 (1.0)	
TR3	24 (20.5)	2 (1.0)		5 (12.5)	0 (0)	
TR4	32 (27.4)	37 (18.7)		13 (32.5)	21 (21.9)	
TR5	33 (28.2)	158 (79.8)		11 (27.5)	74 (77.1)	
ACR-Score 2*^c^*	5 (4-8)	9 (7-10)	<0.001	6 (2-9)	8 (7-10)	<0.001
Rad-score	-0.005 (-1.955-0.910)	1.265 (0.738-1.900)	<0.001	-0.320 (-2.182-0.685)	1.177 (0.355-1.845)	<0.001

Qualitative data were expressed as mean ± standard deviation or number and percentages (%); quantitative data were expressed as median (25%–75% quantiles).

ACR, American College of Radiology; TI-RADS, Thyroid Imaging Reporting and Data System.

^a^ Nodules could have more than one type of echogenic foci.

^b^B-model ultrasound findings based on the senior interpretation.

^c^ACR-Score 1 was referred for the senior radiologist, ACR-Score 2 for the junior radiologist.

### Selecting Radiomics Features and Building the Rad-Score

**Figure 2:**
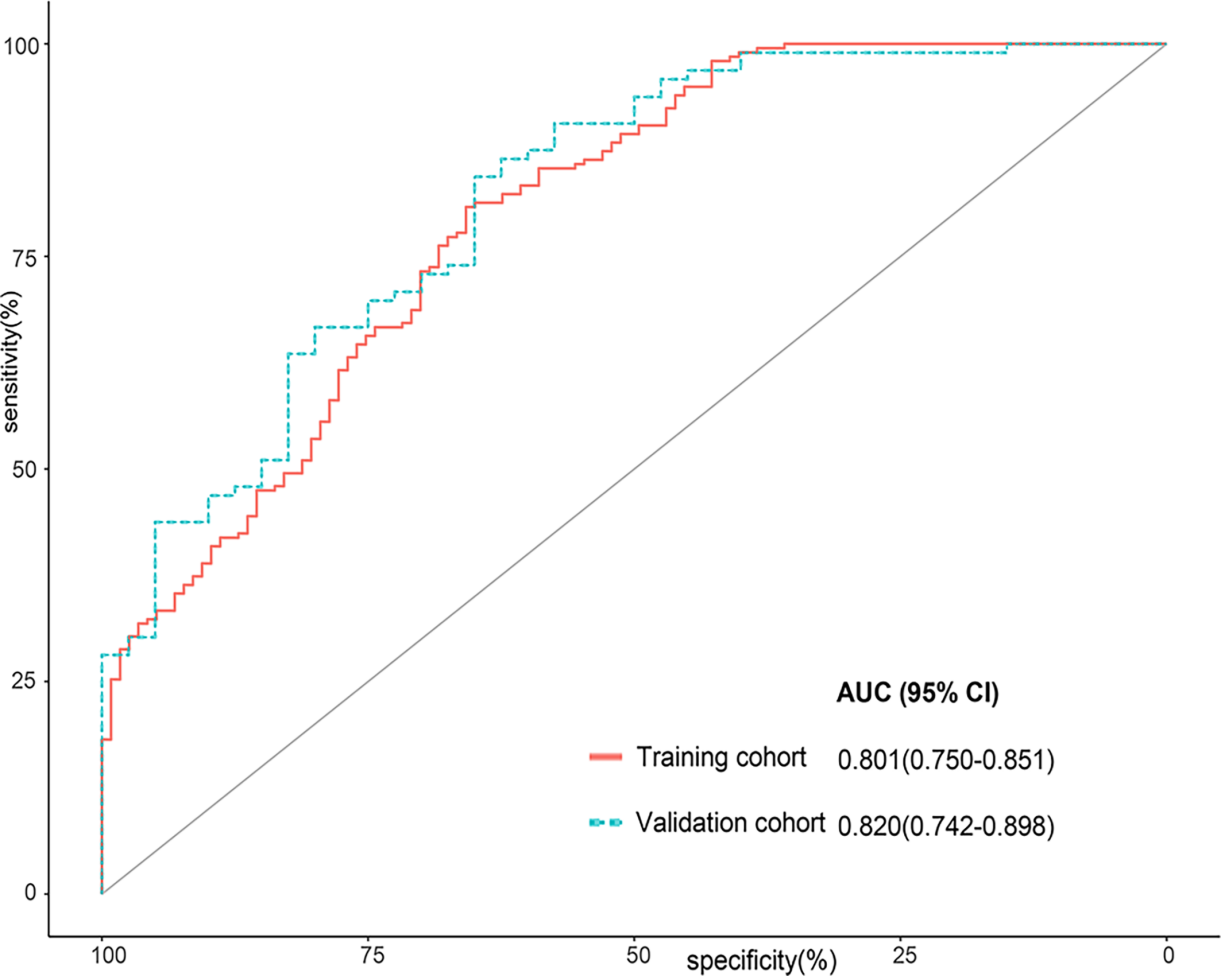
Receiver operator characteristic (ROC) curves for the Rad-Score in the training cohort and the validation cohort.

The rates of intra- and interobserver agreement for the radiomics features reached 94.7% (794/837; mean ICC = 0.920) and 94.0% (787/837; mean ICC = 0.901), respectively (**Supplementary Figure E2**). Seventy-two features were excluded due to unsatisfactory agreement (ICC < 0.75); 192 features were excluded due to insignificant differences based on univariate analysis. Among 14 features selected by LASSO, 4 features were considered to have strong collinearity for the variance inflation factor [VIF] which was more than 10 (**Supplementary Figure E3**). The remaining 10 features were included in the Rad-Score formula (**Supplementary Figure E4**). The Rad-Score of malignant nodules was significantly higher than that of benign nodules in the training [1.265 (0.738–1.900) *vs*. -0.005 (-1.955–0.910), *p* < 0.001] and validation cohorts [1.177 (0.355–1.845) *vs*. -0.320 (-2.182–0.685), *p* < 0.001] (**Table 2**). The Rad-Score yielded an AUC of 0.801 (95% CI 0.750–0.851) in the training cohort and 0.820 (95% CI 0.742–0.898) in the validation cohort (**Figure 2**).

### Development and Performance of the ACR-Rad Nomogram

**Figure 3:**
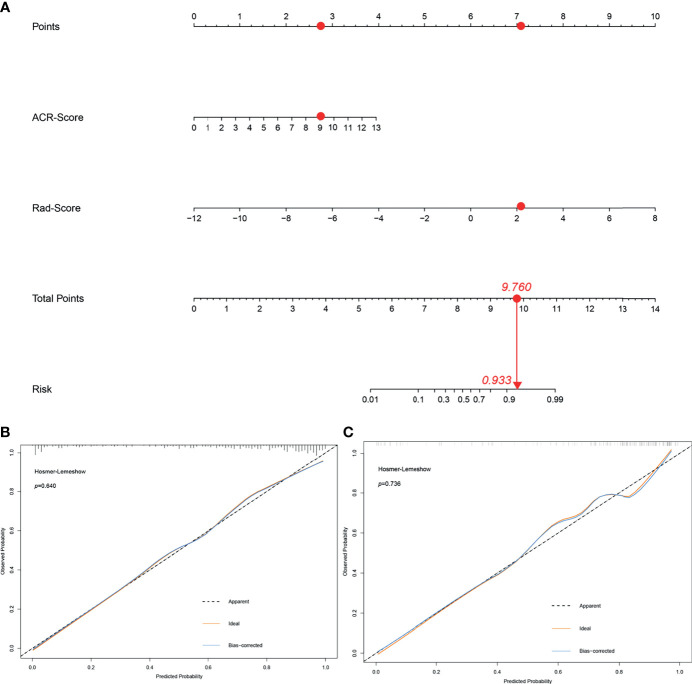
The ACR-Rad nomogram to predict malignancy in thyroid nodules **(A)** and calibration curves of the nomogram in the training **(B)** and validation **(C)** cohort. Red dots described the calculation process of an ACR-Rad nomogram point of a malignant thyroid nodule.

The ACR-Rad nomogram incorporated two predictors: the average ACR-Score [odds ratio (OR) 1.644, 95% CI 1.423–1.928] and Rad-Score (OR 2.269, 95% CI 1.709–3.133) (both *p* < 0.001) (**Figure 3A**). The ACR-Score model was built using the following a univariate logistic regression equation:


$$\text{ACR}-\text{Score model}:\text{logit }\pi_{\text{ACR}-\text{Score}}=-3.791+0.603^{*}\text{ACR}-\text{Score}$$

**Figure 4:**
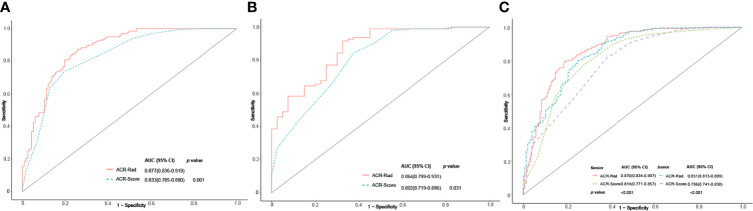
Receiver operating characteristic (ROC) curves of the ACR-Rad nomogram and ACR-Score model in the training **(A)**, validation **(B)**, and entire **(C)** cohorts.

The LR test between the ACR-Rad nomogram and ACR-Score model was χ^2^ = 4.184 (*p* < 0.001). The AIC, BIC, calibration curve, and Hosmer–Lemeshow test statistic (*p* = 0.640) showed good calibration of the ACR-Rad nomogram in the training cohort (**Table 3** and **Figure 3B**). An AUC of 0.877 (95% CI 0.836–0.919) also showed good discrimination, which was significantly higher than that of the ACR-Score model (0.833, 95% CI 0.785–0.880) in the training cohort (*p* < 0.001). The favorable calibration of the nomogram was confirmed in the validation cohort, whose Hosmer–Lemeshow test yielded a *p* value of 0.736 (**Figure 3C**). The AUC (0.864, 95% CI 0.799–0.931) was significantly higher than that of the ACR-Score model (0.802, 95% CI 0.719–0.886) in the validation cohort (**Figures 4A, B**).

**Table 3:** Performance of the ACR-Rad nomogram for predicting malignant thyroid nodules in the training and validation cohorts.

		Multivariate analysis		Discrimination		Calibration	Goodness of fit	
		Odds ratio (95% CI)	*p* value	AUC (95% CI)*^a^*	*p* value	Hosmer–Lemeshow	AIC	BIC
						*p* value*^a^*		
ACR-Rad nomogram	ACR-Score*^b^*	1.644 (1.423-1.928)	<0.001	T: 0.877 (0.836-0.919)/V: 0.864 (0.799-0.931)		T: 0.640/V: 0.736	257.52	268.78
	Rad-Score	2.269 (1.709-3.133)	<0.001					
ACR-Score model		1.827 (1.603-2.114)	<0.001	T: 0.833 (0.785-0.880)/V: 0.802 (0.719-0.886)	0.001/0.031	T: 0.415/V: 0.824	299.43	306.93

ACR, American College of Radiology; AIC, Akaike information criterion; AUC, area under the receiver operating characteristic curve; BIC, Bayesian information criterion; LR, likelihood ratio.

^a^T, training cohort; V, validation cohort.

^b^The average of ACR-Score 1 and ACR-Score 2 was applied.

### Clinical Utility of the ACR-Rad Nomogram

**Figure 5:**
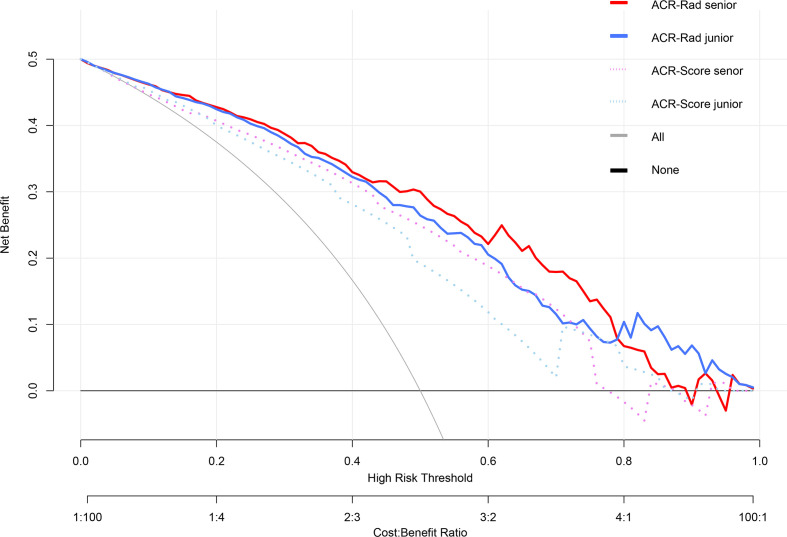
Decision curve analysis (DCA) of the ACR-Rad nomogram in predicting malignancy in thyroid nodules for the senior and junior radiologists. The vertical axis measures standardized net benefit. The horizontal axis shows the corresponding risk threshold. The DCA showed that the ACR-Rad nomogram had a higher overall net benefit than the ACR-Score model for both senior and junior radiologist.

The ICC of ACR-Score (0.677) was considerably lower than that of the Rad-Score and predicted malignancy risk (0.901 and 0.844, respectively). For senior and junior radiologists, the utilization of the ACR-Rad nomogram significantly improved the predictive value for predicting malignant thyroid nodules in terms of AUC, NRI, and IDI compared to the ACR-Score model in entire cohort (all *p <*0.001) (**Table 4** and **Figure 4C**). Moreover, favorable calibration of the nomogram was confirmed in both radiologists (Hosmer–Lemeshow test *p* = 0.715 and 0.415, respectively) (**Supplementary Figure E5**). The DCA demonstrated that the nomogram had a higher overall net benefit than the ACR-Score model, and was more beneficial than either the treat-all or the treat-none strategy (**Figure 5**).

**Table 4:** Performance of the ACR-Rad nomogram for predicting malignant thyroid nodules with interpretations from the senior and junior radiologists.

		AUC (95% CI)	Categorical NRI (95% CI)	Continuous NRI (95% CI)	IDI (95% CI)
ACR-Rad nomogram *vs.* ACR-Score model	For the senior radiologist	0.870 (0.834-0.907) *vs.* 0.814 (0.771-0.857)	0.181 (0.089-0.273)	0.688 (0.505-0.871)	0.121 (0.086-0.155)
	*p* value	<0.001	<0.001	<0.001	<0.001
	For the junior radiologist	0.851 (0.813-0.889) *vs.* 0.786 (0.741-0.830)	0.252 (0.157-0.348)	0.721 (0.539-0.903)	0.138 (0.100-0.175)
	*p* value	<0.001	<0.001	<0.001	<0.001

ACR, American College of Radiology; AUC, area under the receiver operating characteristic curve; IDI, index integrated discrimination improvement; NRI, net reclassification improvement.

When applied with the ACR-Rad_max, specificity, accuracy, and PPV significantly increased with unnecessary FNA rates significantly decreasing but at the expense of significantly decreased sensitivity in comparison to ACR TI-RADS for both senior and junior radiologists. With ACR-Rad_20%/30%/40%, the specificity improved insignificantly for the senior radiologist. With ACR-Rad_50%, the significantly increased specificity, accuracy, and PPV and decreased unnecessary FNA rate were observed for the junior radiologist, and the significantly increased specificity was presented for the senior radiologist as well (all *p* < 0.05), with no difference in sensitivity and negative predictive value (NPV) (*p* > 0.05) (**Table 5**).

**Table 5:** Diagnostic performance and unnecessary FNA rates of ACR TI-RADS and risk cutoff values for the ACR-Rad nomogram.

	ACR	ACR-Rad_20%	ACR-Rad_30%	ACR-Rad_40%	ACR-Rad_50%	ACR-Rad_max
	Senior					
Sensitivity	88.44% (84.22%–91.86%)	96.94% (94.27%–98.59%)*^a^*	94.90% (91.72%–97.12%)*^a^*	89.12% (84.98%–92.44%)	85.03% (80.43%–88.91%)	79.93% (74.89%–84.36%)*^a^*
Specificity	57.96% (49.83%–65.78%)	51.59% (43.49%–59.63%)	59.24% (51.12%–67.00%)	66.24% (58.27%–73.59%)	71.97% (64.26%–78.84%)*^a^*	80.25% (73.16%–86.17%)*^a^*
Accuracy	77.83% (73.71%–81.58%)	81.15% (77.23%–84.66%)	82.48% (78.65%–85.88%)	81.15% (77.23%–84.66%)	80.49% (76.52%–84.05%)	80.04% (76.05%–83.64%)
PPV	79.75% (76.54%–82.63%)	78.95% (76.11%–81.53%)	81.34% (78.28%–84.06%)	83.17% (79.82%–86.07%)	85.03% (81.49%–88.00%)	88.35% (84.62%–91.26%)*^a^*
NPV	72.80% (65.51%–79.04%)	90.00% (82.29%–94.57%)*^a^*	86.11% (78.83%–91.17%)*^a^*	76.47% (69.70%–82.12%)	71.97% (65.79%–77.43%)	68.11% (62.66%–73.10%)
Unnecessary FNA rate	20.25% (16.02%–25.02%)	21.05% (16.96%–25.63%)	18.66% (14.68%–23.19%)	16.83% (12.86%–21.42%)	14.97% (11.09%–19.57%)	11.65% (8.06%–16.13%)*^a^*
	Junior					
Sensitivity	90.88% (87.01%–93.90%)	97.62% (95.16%–99.04%)*^a^*	95.92% (92.98%–97.87%)*^a^*	92.86% (89.29%–95.52%)	87.07% (82.69%–90.69%)	80.27% (75.26%–84.67%)*^a^*
Specificity	38.85% (31.19%–46.95%)	46.50% (38.51%–54.62%)	52.87% (44.75%–60.87%)*^a^*	57.96% (49.83%–65.78%)*^a^*	64.33% (56.30%–71.81%)*^a^*	74.52% (66.96%–81.13%)*^a^*
Accuracy	72.85% (68.50%–76.89%)	79.82% (75.82%–83.43%)*^a^*	79.21% (76.32%–81.83%)*^a^*	80.71% (76.76%–84.25%)*^a^*	79.16% (75.11%–82.82%)*^a^*	78.27% (74.17%–81.99%)
PPV	73.70% (71.11%–76.14%)	77.36% (74.68%–79.83%)	87.37% (79.58%–92.47%)	80.53% (77.44%–83.29%)*^a^*	82.05% (78.67%–85.00%)*^a^*	85.51% (81.78%–88.58%)*^a^*
NPV	69.32% (60.00%–77.29%)	91.25% (83.11%–95.67%)*^a^*	80.93% (77.00%–84.45%)*^a^*	81.25% (73.75%–86.98%)	72.66% (65.90%–78.52%)	66.86% (61.15%–72.11%)
Unnecessary FNA rate	26.45% (21.98%–31.30%)	22.64% (18.48%–27.24%)	20.79% (16.69%–25.38%)	19.47% (15.39%–24.09%)*^a^*	17.95% (13.85%–22.67%)*^a^*	14.49% (10.56%–19.21%)*^a^*

^a^A statistically significant difference.

FNA, fine-needle aspiration; NPV, negative predictive value; PPV, positive predictive value.

## Discussion

In this study, we proved that BMUS radiomics and the ACR-Rad nomogram based on it and ACR TI-RADS can accurately predict malignancy in thyroid nodules, and the nomogram showed significantly better discrimination and calibration performance than the guideline alone. Excellent repeatability and clinical application of the nomogram were demonstrated in the entire cohort. With performing with 50% risk cutoff, the nomogram increased the specificity, accuracy, and PPV and decreased the unnecessary FNA rates of ACR TI-RADS for radiologists of different proficiency levels.

Predicting malignant thyroid nodules and reduction in the number of meaningless biopsies are original intentions of many guidelines that the ACR TI-RADS can meet. The ACR guideline assigns points to the five feature categories of BMUS. The sum of the points is used to determine the probability of malignancy and provides recommended management procedures (16). However, the clinical application of the ACR guideline is strongly subjective (6, 13, 14). Hoang et al. (6) found that when the judgment of composition was wrong, malignant nodules would be misclassified. Although the ACR risk stratification system is fault-tolerant, in our study, the interobserver agreement was unsatisfactory (ICC = 0.677) and lower than that of the Rad-Score and risk prediction value of the nomogram (ICC = 0.901 and 0.844, respectively). The reason may be due to weaker judgment of the junior radiologist in scoring spongiform, very hypoechoic and ill defined. Furthermore, the specificity of the ACR TI-RADS is weak (38.85%–57.96% in our study).

Previous studies have reported that combining clinical characteristics (such as age, thyrotropin, or sex) with ultrasound features (such as ACR TI-RADS lexicon, hypoechoic halo, or blood flow) slightly increased the accuracy of these models in discriminating malignant nodules from benign nodules than risk stratification systems (23, 24). However, the abovementioned clinical characteristics in the study of Liang et al. (13) were not significantly different. In our study, there was no significant difference in the gender. Moreover, other subjective ultrasound features might make little contribution to solve current challenges.

With the recent development of radiomics, its application in predicting the malignancy of thyroid nodules has received attention. Previous studies reported that radiomics showed good performance in predicting thyroid cancer, which was even higher than the risk classification guidelines with interpretations from non-experts (13, 25, 26). In our study, both the ACR-Score and Rad-Score were independent predictive factors of malignant nodules, and the Rad-Score had favorable diagnostic performance in magnificent nodules. However, radiomics alone cannot improve the performance of the ACR TI-RADS for senior radiologists who are experienced to evaluate comprehensively ultrasound features correlated with properties of the nodules (15).

Park et al. (14) demonstrated that when combined with a 5% predicted malignancy risk cutoff of radiomics with the ACR or American Thyroid Association (ATA) guidelines, the performance significantly increased and unnecessary FNA rates reduced; in consequence, combining radiomics with ultrasound-based risk stratification systems is a potential approach to predict magnificent thyroid nodules. Luo et al. (27) constructed a nomogram including the Rad-Score and feature categories of the ACR TI-RADS and determined that a combination model was better than radiomics and the ACR TI-RADS alone for discriminating benign and malignant thyroid nodules. In our study, the ACR-Rad nomogram could be a more convenient tool to combine the ACR-Score and Rad-Score and a better predictive model for thyroid cancer. For senior and junior radiologists, the nomogram had significantly improved predictive performance in comparison with the ACR TI-RADS.

The radiomics model has not been sufficiently evaluated by prior studies, which were limited to comparisons of discrimination performance or only had senior radiologists assigned to score and delineate nodules (28, 29). Our study evaluated the repeatability, discrimination, and clinical utilization of the ACR-Rad nomogram applied by senior and junior radiologists, proving that there was strong consistency in processing nodule texture information and it significantly increased the predictive performance among radiologists of different proficiency levels, which can compensate for the relatively low repeatability and accuracy of ACR TI-RADS. Moreover, the appropriate cutoff of the ACR-Rad nomogram can significantly reduce unnecessary FNA rates, increase the specificity and PPV, and maintain the high sensitivity of the guideline especially for junior radiologists.

Our study had several limitations. First, this study was a single-center retrospective study; thus, selection bias may be inevitable. The proportion of benign nodules in this study was much lower than that in other studies (13–15), because we chose nodules with postoperative pathology instead of FNA or follow-up. Second, BMUS images were only acquired with Philips ultrasound instruments. We should investigate the influence from images of different ultrasound instruments. Third, on account of the overlap between the shape features and the ACR TI-RADS, we did not analyze them. The predictive performance of shape features should be explored further.

In conclusion, the ACR-Rad nomogram, combined with ACR TI-RADS and BMUS radiomics, has the potential to be a convenient and accurate tool to predict malignancy and improve performance for radiologists at different proficiency levels in thyroid nodules.

## Data Availability Statement

The original contributions presented in the study are included in the article/**Supplementary Material**. Further inquiries can be directed to the corresponding author.

## Ethics Statement

Ethical review and approval was not required for the study on human participants in accordance with the local legislation and institutional requirements. The requirement for informed consent was waived. Written informed consent was obtained from the individual(s) for the publication of any potentially identifiable images or data included in this article.

## Author Contributions

XH performed the formal analysis, contributed to the methodology, administered the project, and wrote the original draft of the manuscript. ZW curated the data and collected the resources. AZ conceived the study and wrote and reviewed the manuscript. XM curated the data. QQ curated the data. CZ curated the data. SC curated the data. PX supervised the study and wrote, reviewed, and edited the manuscript. All authors contributed to the article and approved the submitted version.

## Funding

This study was supported by “the key research and development projects of Jiangxi Province” (no. 20181BBG70031).

## Conflict of Interest

The authors declare that the research was conducted in the absence of any commercial or financial relationships that could be construed as a potential conflict of interest.

## Publisher’s Note

All claims expressed in this article are solely those of the authors and do not necessarily represent those of their affiliated organizations, or those of the publisher, the editors and the reviewers. Any product that may be evaluated in this article, or claim that may be made by its manufacturer, is not guaranteed or endorsed by the publisher.
