# Supplementary material for: Nomogram Combining Radiomics With the American College of Radiology Thyroid Imaging Reporting and Data System Can Improve Predictive Performance for Malignant Thyroid Nodules
Source: Front Oncol. 2021 Oct 13;11:737847. doi: 10.3389/fonc.2021.737847 (PMC8550451; doi:10.3389/fonc.2021.737847)
Supplement: Supplementary file 1 [file DataSheet_1.pdf]

SUPPLEMENTARY MATERIAL

A1 Resampling and Z-score normalization

we adopted resampling as a preprocessing method, which was performed to obtain a voxel size of 1 x 1 x 1 mm^3 via trilinear interpolation before feature calculation.

Different radiomics features have different value ranges, which makes it difficult to compare two features with variable orders of magnitude. The z-score normalization was employed to eliminate different feature magnitudes by scaling values to a mean of 0 and a standard deviation of 1 using the following formula:

$$z = \frac{x-\mu}{\sigma}$$

where μ is the mean for the population and σ is the standard deviation for the population.

A2 Detailed methodology to extract radiomics features

To avoid the curse of dimensionality and reduce the bias from radiomics features when modeling, four steps were adopted to select the features in the training cohort. First, inter-observer and intra-observer agreement of radiomics features indicated dissatisfactory agreement (ICC$\leqslant$0.75) were excluded. Second, the independent samples *t* test or Mann-Whitney *U* test was performed on radiomics features, which did not meet either of the above tests were excluded. Third, least absolute shrinkage and selection operator (LASSO) was performed for dimensionality reduction and feature selection by performing variable selection and regularization to enhance the prediction accuracy and interpretability of the statistical model produced. Adjust the regularization parameter (λ) with the minimum criteria, and select features with 10-fold cross-validation. Finally, the variance inflation factors (VIF) for the features selected by LASSO were calculated, and the features of VIF more than 10 were excluded to avoid severe linear dependence.

Supplementary Tables

Supplementary Table 1. Five feature categories in American College of Radiology (ACR) Thyroid Imaging Reporting and Data System (TI-RADS) lexicon

score	Composition	Echogenicity	Shape	Margin	Echogenic foci
0	Cystic or spongiform	Anechoic	Taller-than-wide	Smooth or ill defined	No echogenic foci or Large comet tail
1	Cystic and solid	Hyper- or Isoechoic	-	-	Macrocalcifications
2	Solid	Hypoechoic	-	Irregular or lobulated	Peripheral
3	-	Very hypoechoic	Not taller-than-wide	Extrathyroidal extension	Punctate

Supplementary Table 2. American College of Radiology Thoracic Imaging Reporting and Data System risk stratification system and management recommendations

Category	US features	Follow size cutoff	FNA size cutoff
Highly suspicious	7 points or more	0.5 cm	1 cm
Moderately suspicious	4 to 6 points	1.0 cm	1.5 cm
Mildly suspicious	3 points	1.5 cm	2.5 cm
Not suspicious	2 points	Not indicated	Not indicated
Benign	0 points	Not indicated	Not indicated

FNA = fine-needle aspiration.

Supplementary Table 3. List of radiomics features

Radiomics feature Group	Radiomics features
Gray Level Dependence Matrix (GLDM)	GrayLevelVariance, HighGrayLevelEmphasis, DependenceEntropy, DependenceNonUniformity, GrayLevelNonUniformity, SmallDependenceEmphasis, SmallDependenceHighGrayLevelEmphasis, DependenceNonUniformityNormalized, LargeDependenceEmphasis, LargeDependenceLowGrayLevelEmphasis, DependenceVariance, LargeDependenceHighGrayLevelEmphasis, SmallDependenceLowGrayLevelEmphasis, LowGrayLevelEmphasis
Gray Level Co-occurrence Matrix (GLCM)	JointAverage, SumAverage, JointEntropy, ClusterShade, MaximumProbability, Idmn, JointEnergy, Contrast, DifferenceEntropy, InverseVariance, DifferenceVariance, Idn, Idm Correlation, Autocorrelation, SumEntropy, MCC, SumSquares, ClusterProminence, Imc2, Imc1, DifferenceAverage, Id, ClusterTendency
First Order	InterquartileRange, Skewness, Uniformity, Median, Energy, RobustMeanAbsoluteDeviation, MeanAbsoluteDeviation, TotalEnergy, Maximum, RootMeanSquared, 90Percentile, Minimum, Entropy, Range, Variance, 10Percentile, Kurtosis, MeanInterquartileRange, Skewness, Uniformity, Median, Energy, RobustMeanAbsoluteDeviation, MeanAbsoluteDeviation, TotalEnergy, Maximum, RootMeanSquared, 90Percentile, Minimum, Entropy, Range, Variance, 10Percentile, Kurtosis, Mean

Gray Level Run Length Matrix (GLRLM)	ShortRunLowGrayLevelEmphasis, GrayLevelVariance, LowGrayLevelRunEmphasis, GrayLevelNonUniformityNormalized, RunVariance, GrayLevelNonUniformity, LongRunEmphasis, ShortRunHighGrayLevelEmphasis, RunLengthNonUniformity, ShortRunEmphasis, LongRunHighGrayLevelEmphasis, RunPercentage, LongRunLowGrayLevelEmphasis, RunEntropy, HighGrayLevelRunEmphasis, RunLengthNonUniformityNormalized
Gray Level Size Zone Matrix (GLSZM)	GrayLevelVariance, ZoneVariance, GrayLevelNonUniformityNormalized, SizeZoneNonUniformityNormalized, SizeZoneNonUniformity, GrayLevelNonUniformity, LargeAreaEmphasis, SmallAreaHighGrayLevelEmphasis, ZonePercentage, LargeAreaLowGrayLevelEmphasis, LargeAreaHighGrayLevelEmphasis, HighGrayLevelZoneEmphasis SmallAreaEmphasis, LowGrayLevelZoneEmphasis, ZoneEntropy, SmallAreaLowGrayLevelEmphasis
Neighbouring Gray Tone Difference Matrix (NGTDM)	Coarseness, Complexity, Strength, Contrast, Busyness
Wavelet	HHH, HLL, HLH, HHL, LLH, LHL, LHH, LLL

Supplementary Table 4. Major packages of R software used in this study

Functions	R package
LASSO regression and univariate logistic regression analysis	glmnet

Measure the area under the receiver operating curve (AUC), akaike information criterion(AIC) and delong test	pROC
Plot bar diagrams, correlation coefficient matrix and ROC	ggplot2
Plot nomogram	Hmisc, regplot
Plot calibration curves	rms
Index integrated discrimination improvement(IDI) and net reclassification improvement (NRI)	PredictABEL
Bayesian information criterion(BIC)	nlme
Hosmer-Lemeshow test	ResourceSelection
Decision curve analysis (DCA)	rmda

Supplementary Figures

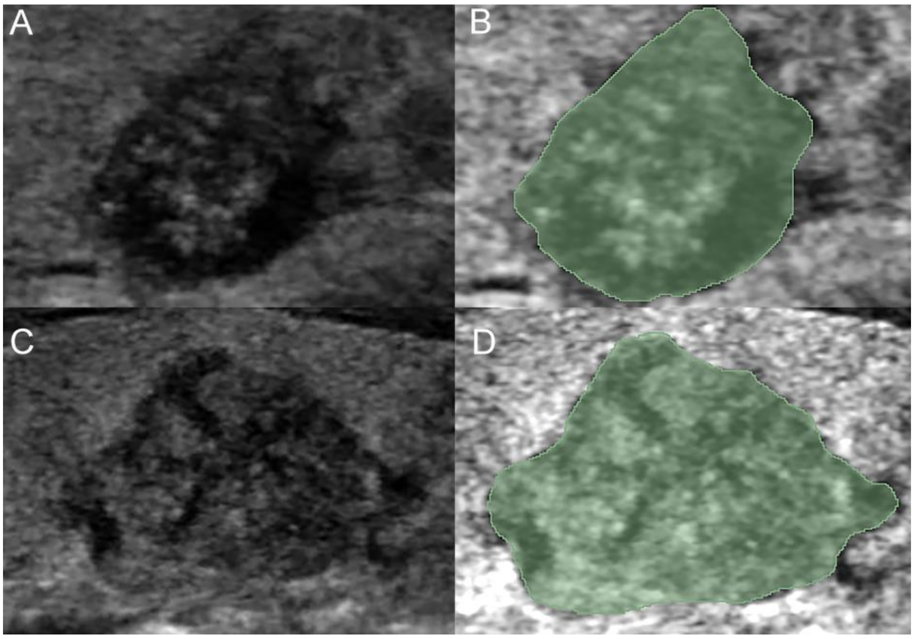

Supplementary Figure 1 B-mode ultrasound and region of interest (ROI) images of thyroid nodules. A A papillary thyroid cancer in a 50-year-old female patient is 8x10-mm in diameter, and its ACR-Score 1 is 10, ACR-Score 2 is 12, Rad-score is

2.052; **B** The green region shows ROI. **C** A nodular goiter in a 56-year-old female nodule is 13x8-mm in diameter, and its ACR-Score 1 is 9, ACR-Score 2 is 9, Rad-score is 0.755; **D** The green region shows ROI.

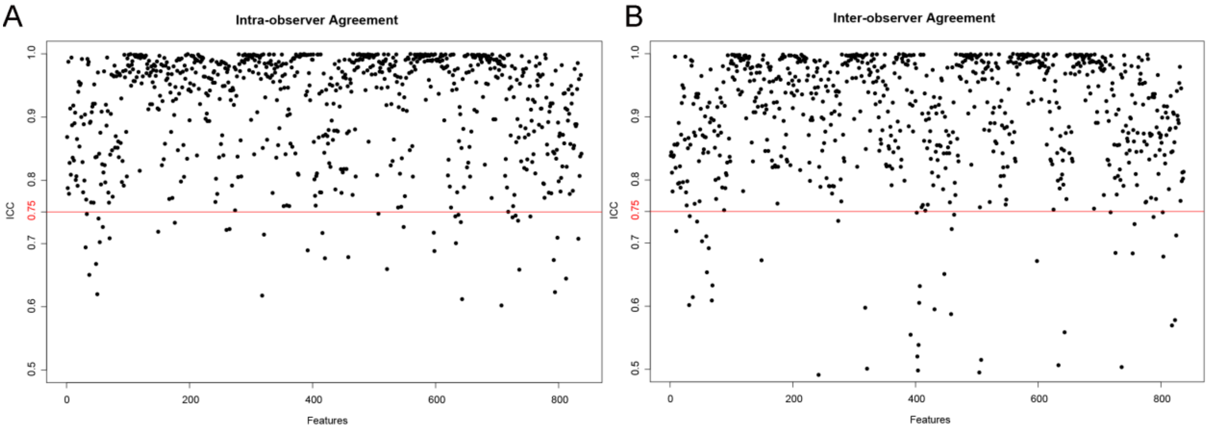

Supplementary Figure 2 Evaluation of feature stability and inter-observer and intra-observer agreement based on the interclass correlation coefficient (ICC). A 94.7% (794/837) features presented good intra-observer agreement with ICCs of >0.75 (above the red cutoff line). **B** 94.0% (787/837) features presented good intra-observer agreement with ICCs of >0.75 (above the red cutoff line).

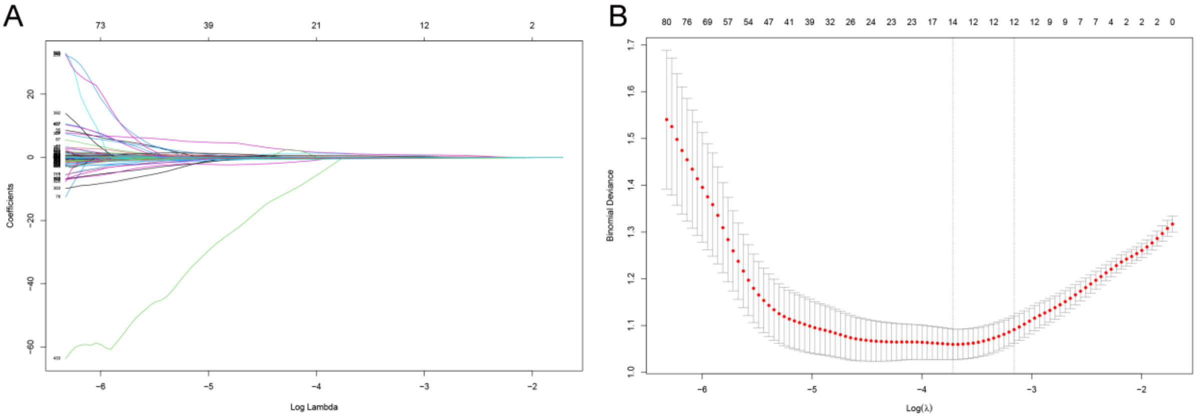

Supplementary Figure 3 B-mode ultrasound (BMUS) image feature selection using the least absolute shrinkage and selection operator (LASSO) logistic regression model in the training cohort. A LASSO coefficient profiles of the BMUS for nodular radiomics features. **B** The 10-fold cross-validation and the minimal criteria process was used to generate the optimal penalization coefficient

lambda (λ) in the LASSO model. As a result, λ values of 0.024 was selected. Dotted lines on the left and right denote the minimum criterion and 1-standard error criterion (1-SE), respectively. The minimum criterion was applied.

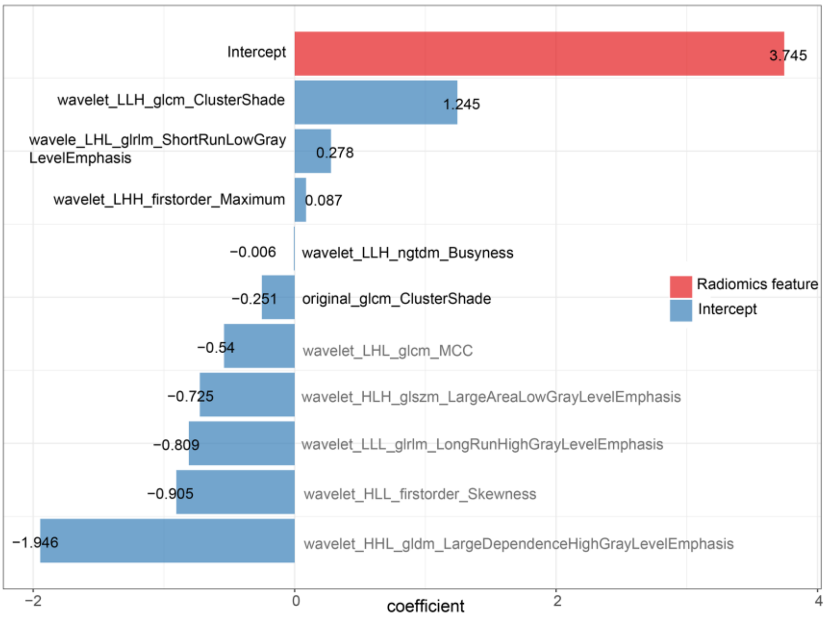

Supplementary Figure 4 Histogram showing the coefficients of the selected features in the Rad-Score formula.

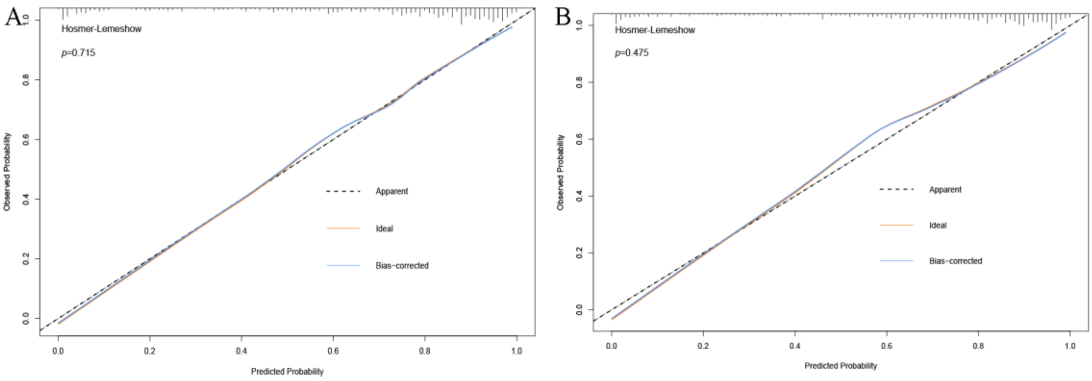

Supplementary Figure 5 Calibration curves of the ACR-Rad nomogram for the senior (A) and junior radiologist (B) in entire cohort.
